# Modeling and Simulation of Multi-Pulse Femtosecond Laser Ablation of WC-6Co Cemented Carbide

**DOI:** 10.3390/mi17010011

**Published:** 2025-12-23

**Authors:** Jin Wang, Haijiao Xu, Shiwei Zhang, Lianyu Fu

**Affiliations:** 1 School of Mechanical Engineering, University of Science and Technology Beijing, Beijing 100083, China; m202320679@xs.ustb.edu.cn (H.X.); zsw139304@126.com (S.Z.); 2 Shenzhen Jinzhou Precision Technology Co., Ltd., Shenzhen 518100, China; mlyfu@163.com

**Keywords:** femtosecond laser, cemented carbide, multi-pulse simulation modeling, ablation effect

## Abstract

In the process of femtosecond pulsed laser machining of cemented carbide, the simulation study of multi-pulse laser ablation is of great significance in revealing the laser ablation effect and guiding the machining process. When performing modeling, the consideration of the two-temperature equation, cumulative effect, and the improvement in the energy attenuation term in the laser heat source have a great influence on the simulation accuracy, but there are few related studies. This study aims to solve the above three problems and establish a more accurate finite element simulation model to reveal the effect of femtosecond laser ablation of cemented carbide. In order to apply the dual-temperature equation, the thermophysical parameters are chosen to approximate similar materials through experiments; in order to consider the cumulative effect, the accumulation coefficients are obtained through experiments and corrected based on the energy accumulation model for the laser heat source. In order to consider the influence of multi-pulse laser processing on the energy decay term in the traditional laser body heat source, the energy decay term is expanded by transforming the starting surface of the attenuation. The multi-pulse laser two-temperature model is established based on the WC-6Co material, the temperature field distribution and ablation morphology of the sample surface under the action of femtosecond laser are obtained, and the accuracy of the simulation model is verified by experimental results. On this basis, the influence of the average power and the number of pulses of the multi-pulse laser on the ablation effect of the material is quantitatively investigated.

## 1. Introduction

Cemented carbide is widely used in micro-tools due to its high machining precision. However, conventional mechanical grinding is limited by the material’s high hardness and low toughness, which often cause tool deformation or fracture during miniaturization. In contrast, femtosecond laser machining offers a non-contact solution ideal for such hard materials, enabling high surface quality. Despite this potential, the ultrafast and transient nature of femtosecond ablation involves complex microscopic processes that are difficult to capture experimentally. Consequently, numerical modeling is essential to elucidate the underlying ablation mechanisms and laser–material interactions.

Existing simulations of laser ablation on cemented carbide have predominantly relied on Fourier’s heat conduction law, focusing on the nanosecond regime. For instance, Yilbas et al. [1] and C.K. et al. [2] demonstrated that steep temperature gradients induce significant thermal stresses, leading to subsurface cracking. Similarly, Yao et al. [3] and Zhang et al. [4] utilized these macroscopic models to correlate laser parameters with heat-affected zones (HAZs) and damage mechanisms. However, the validity of Fourier’s law depends on thermal equilibrium between electrons and the lattice, an assumption that holds only for pulse widths in the nanosecond range or longer [5]. For femtosecond lasers, this equilibrium breaks down due to the ultrafast energy deposition. Consequently, traditional macroscopic models fail to capture the transient electron–lattice non-equilibrium, rendering them unsuitable for simulating femtosecond ablation.

Soviet scholar Anisimov first proposed the two-temperature model (TTM) to describe the interaction between ultrashort pulsed lasers and metals [6]. Detlef et al. [7] demonstrated that the TTM effectively captures the temperature evolution of electrons and the lattice on picosecond timescales. Given the highly non-equilibrium thermodynamic state during ablation, describing these subsystems using the TTM is essential [8,9]. In terms of specific applications, K.J.C. et al. [10] developed a one-dimensional axisymmetric TTM for metal thin films. They analyzed superheating phenomena and found that higher laser fluence leads to larger ablation depths. However, they noted that the simplified 1D model tends to overestimate temperature responses by neglecting radial heat transfer. Li et al. [11] extended this to a three-dimensional TTM to investigate the femtosecond ablation of aluminum films. They successfully visualized the energy conversion process and 3D ablation pit morphologies, confirming the model’s reliability through experimental validation. Furthermore, Hu [12] utilized the TTM to simulate multi-burst picosecond laser ablation on metals and ceramics. The study revealed that ablation depth in multi-burst modes depends heavily on the pulse interval, and energy accumulation between neighboring pulses significantly enhances ablation efficiency. Similarly, Wang [13] employed COMSOL Multiphysics to establish a TTM for Ti6Al4V alloy. By using a phase indicator, this study obtained the electron–lattice temperature distribution and accurately predicted the dimensions of the ablation morphology. In summary, while the TTM has been widely used to simulate ultrashort-pulse laser ablation of various materials, research specifically targeting cemented carbide remains scarce. Therefore, it is essential to apply this framework to investigate the ablation behavior of cemented carbide.

In practical applications, multiple pulses are often required for effective ablation, where subsequent pulses interact with modified material, altering the ablation threshold. This cumulative effect [14] must therefore be incorporated into simulations. Cai et al. [15] established a heat conduction model considering evaporation, plasma shielding, and accumulation to demonstrate simultaneous shaping and sharpening. Building on this framework, Song et al. [16] optimized process parameters, identifying a specific power density range for ideal processing. Similarly, Lin et al. [17] investigated multi-pulse femtosecond ablation of 18Cr2Ni4WA gear steel. By establishing an energy absorption model that accounts for accumulation, they quantified the relationship between crater dimensions and pulse count, determining the optimal power settings for quality processing. Ming et al. [18] addressed energy accumulation and defocusing effects on tooth surfaces by developing a complex coupling model. Using the finite-difference method, they calculated electron–lattice temperature evolution and simulated pit geometry. By incorporating variations in spot energy distribution at different incident angles into this model, a comprehensive simulation was established. Furthermore, by inputting the angle between the workpiece surface and the horizontal plane, required laser parameters could be determined to improve efficiency. Despite these advances, simulation studies on cemented carbide remain limited. D. Metzner et al. [19] modeled the high-frequency ablation of cemented carbide, finding that thermal accumulation significantly enhances material removal volume. However, this study did not integrate the two-temperature model with cumulative effects to simulate the femtosecond ablation of cemented carbide, a gap this study aims to fill.

In multi-pulse processing, the effective laser interaction surface continuously evolves due to the increasing depth of the ablation pit. Consequently, the standard volumetric heat source model based on the Beer–Lambert law requires modification to account for this dynamic geometry. However, few numerical simulations have addressed this aspect.

For instance, Hu [12] utilized a two-temperature model to analyze energy transfer in multi-pulse modes. The study attributed the increased ablation volume per pulse to enhanced electron–phonon coupling and energy accumulation, a finding verified by experiments. Caterina et al. [20] simulated burst-mode irradiation on stainless steel. By analyzing the electron–lattice temperature fields, they found that the ablation threshold fluence decreases as the number of sub-pulses increases. Furthermore, Jing et al. [21] developed a hybrid model for nanosecond–picosecond double-pulse laser processing of CFRP, combining a volumetric heat source for the picosecond pulse with a surface heat source for the nanosecond pulse to simulate the material removal process. Despite these advancements, most studies have not updated the energy attenuation term to reflect the changing crater depth. From this perspective, improving the energy attenuation description is critical to enhancing the accuracy of multi-pulse laser simulation models.

In summary, accurate simulation of femtosecond multi-pulse laser ablation of WC-6Co requires a two-temperature model that accounts for cumulative effects and incorporates an improved energy attenuation term. Therefore, this study first establishes a comprehensive two-temperature model for the femtosecond laser ablation of WC-6Co cemented carbide using COMSOL Multiphysics. To ensure model fidelity, thermophysical parameters are approximated based on similar substances, while cumulative effects and a refined energy attenuation term are integrated to accurately characterize the ablation process. Subsequently, the finite element model is verified through laser ablation experiments. Finally, based on the validated model, the influence of average laser power and pulse number on the ablation outcomes is systematically analyzed.

## 2. Femtosecond Laser Experimental Setup

### 2.1. Femtosecond Laser and Optical Path System

**Figure 1:**
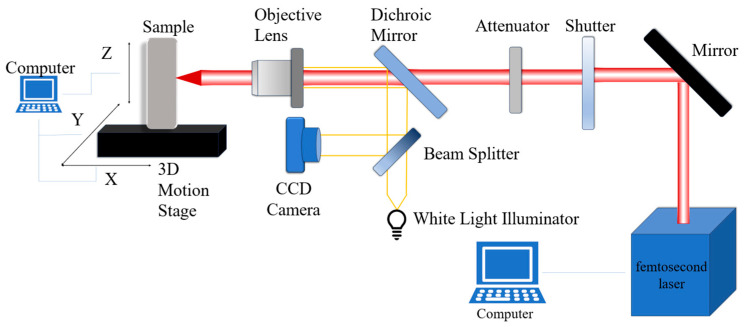
Laser Manufacturing Optical Path System.

Figure 1 illustrates the optical setup of the laser manufacturing system. A Yb:KGW femtosecond laser amplifier (Pharos, Light Conversion Co., Ltd., Shenzhen, China) with a central wavelength of 1030 nm was employed as the Gaussian source. The laser operated at a repetition rate of 1 kHz and a pulse width of 400 fs. Mirrors were used to steer the beam into a shutter, which regulated the on–off state of the optical path. This allowed for precise control over the irradiation time and the ablation process. To adjust the incident laser power, an attenuator consisting of a half-wave plate and a polarizing beam splitter was utilized. Subsequently, a 40× objective lens focused the modulated beam onto a micro-area of the sample surface. The WC-6Co sample was mounted on a computer-controlled three-dimensional motion stage. During the machining process, the stage moved within the XY plane to facilitate laser removal. Additionally, a monitoring system utilizing a dichroic mirror was integrated into the setup to strictly control the focal position on the sample surface.

### 2.2. Measuring Devices

The grain size of the material before processing was characterized using a cold-field emission scanning electron microscope (SEM, JEOL JSM-6701F) manufactured by JEOL Ltd., Akishima, Japan. The polishing of the finely ground samples was performed using a Mecatech Z64 polishing machine produced by PRESI, Eybens, France. Surface roughness was measured by a white-light interferometer (WLI, Rtec-WLI1000) from RTEC Instruments (San Jose, CA, USA). The sample used was YG6 grade with model number XC161008, produced by Zhuzhou Diamond Cutting Tools Co., Ltd. (Zhuzhou, China), with dimensions of 15 mm × 15 mm × 4 mm. The material surface was pretreated by polishing, exhibiting a grain size of 1.75 µm ad a density of 14.9 g/cm^3^.

Femtosecond laser ablation involves minimal material removal, producing micrometer-scale craters with nanometer-scale depths. Consequently, high surface smoothness is essential for accurate experimental observation. Prior to experimentation, samples were mechanically polished to a surface roughness (Ra) of 0.015 µm. They were then ultrasonically cleaned and dried with cold air. Following laser processing, the samples underwent a 20 min ultrasonic cleaning and low-temperature drying. The ablation diameter and morphology were characterized using a Keyence VH-X7000 digital microscope (Osaka, Japan). Additionally, ablation depths were measured via a white-light interferometer (WLI, Rtec-WLI1000, San Jose, CA, USA). To ensure data reliability, multiple trials were conducted to mitigate measurement fluctuations. The arithmetic mean of these results was used for subsequent analysis.

## 3. Femtosecond Laser Multi-Pulse Ablation Modeling

**Figure 2:**
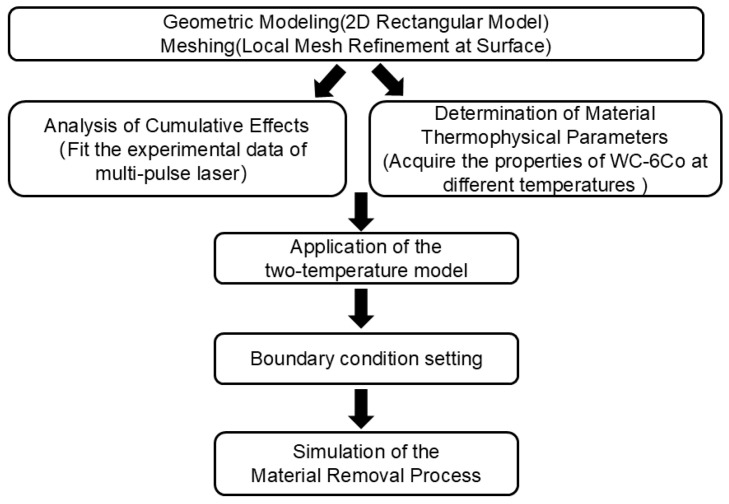
Logic flow chart of the modeling procedure.

In this study, a numerical model for multi-pulse femtosecond laser ablation of cemented carbide is established based on the finite element method. To facilitate understanding of the calculation sequence, the logic flow of the modeling procedure is briefly summarized in Figure 2.

### 3.1. Geometric Modeling and Meshing

The laser processing process is complex and influenced by many factors, making it challenging to establish a mathematical model that fully reflects reality. Therefore, to facilitate modeling and ensure that the simulation more accurately represents actual conditions, the following reasonable assumptions were made in this study.

(1) The laser beam used has an ideal Gaussian distribution in both the spatial and temporal domains.
(2) The workpiece material is homogeneous and isotropic.
(3) The surface of the workpiece is considered smooth and flat.
(4) The material does not absorb part of the laser light that has been reflected out.

It is important to acknowledge the limitations imposed by the assumption of material homogeneity and isotropy. In reality, cemented carbide is a heterogeneous composite consisting of hard WC grains embedded in a metallic Co binder, which possess distinct thermophysical and optical properties. By treating the material as a continuous medium with effective properties, the current model focuses on the thermal evolution and ablation morphology. The primary limitation of this approach lies in its inability to model more intricate phenomena. Consequently, Simulations predict smooth ablation profiles, whereas experimental results typically exhibit more complex and irregular ablation profiles. However, for the purpose of predicting key dimensions (e.g., depth and diameter) and optimizing process parameters, this simplified approximation provides a necessary balance between computational efficiency and predictive accuracy. In future work, we intend to conduct a systematic study building upon the proposed model, specifically incorporating material heterogeneity and anisotropy into the simulation predictions.

Considering the symmetric nature of the Gaussian pulse, a two-dimensional rectangular model is adopted in the simulation instead of a three-dimensional one. Based on the actual dimensions of the tungsten carbide sheets used in the experiments, it is assumed that all surfaces—except the top surface—remain at room temperature during ablation. Therefore, the material is treated as infinitely thick in both the transverse and longitudinal directions.

**Figure 3:**
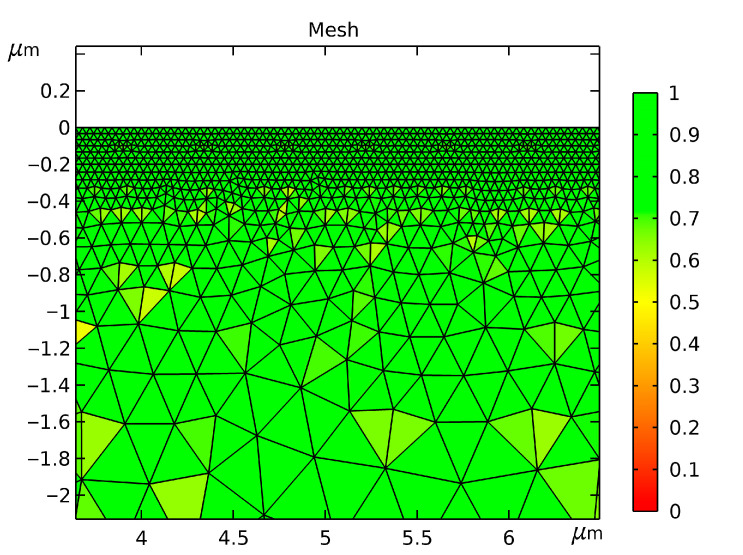
Mesh Generation for the Multi-Pulse Laser Ablation Model.

In femtosecond multi-pulse laser processing, the thermal penetration depth is typically limited to the nanometer scale. Consequently, a highly refined mesh is essential at the laser-irradiated surface. To balance accuracy and efficiency, a two-dimensional linear element mesh was used to discretize the geometric model. Boundary refinement was implemented via a customized distribution scheme. Specifically, boundary element sizes were constrained between 0.01 µm and 0.04 µm, while the locally refined region utilized sizes ranging from 0.002 µm to 0.015 µm. Figure 3 depicts the mesh layout at the laser–material interaction zone. The coordinates are expressed in micrometers, with the incident laser spot centered at (x_0_ = 5, y_0_ = 0). As indicated by the color scale and numerical values, a value approaching 1 signifies superior mesh quality.

### 3.2. Cumulative Effects

For metals, this cumulative effect behavior is due to the accumulation of plastic deformations due to the laser-induced thermal stress field [22]. P.T. Mannion et al. [22] proposed that the decrease in ablation threshold with the number of incident laser pulses follows a power-law relationship, which is applicable to semiconductors, insulators, and metals. In this context, the relationship between the damage threshold *F*_th_(*N*) after *N* pulses and the single-pulse damage threshold $F_{th}\left(1\right)$ can be expressed as:

(1) $$F_{th}\left(N\right)=F_{th}\left(1\right)N^{S-1}$$

where *S* is the cumulative coefficient, used to describe the degree of cumulative effect in the material; when *S* is 1, there is no cumulative effect in the material.

Multiplying both sides of Equation (1) by *N* and then taking the logarithm of each collapses to give:

(2) $$\ln\left[NF_{th}\left(N\right)\right]=SlnN+lnF_{th}\left(1\right)$$

Equation (2) shows that $\ln\left[NF_{th}\left(N\right)\right]\;$ is linearly related to lnN, and the slope is the cumulative coefficient S. Therefore, in order to obtain the cumulative coefficient *S*, it is necessary to know the damage threshold ${\;F}_{th}\left(N\right)\;$ at each pulse number.

According to Mannion P et al. [22] mentioned the relationship between the multi-pulse ablation diameter $D\left(N\right)$ (μm) and $F_{th}\left(N\right)$.

(3) $$D^{2}\left(N\right)=2r_{0}^{2}\left(\ln P+\ln\frac{2}{\pi r_{0}^{2}fF_{th}\left(N\right)}\right)$$

where *N* is the number of pulses, $D\left(N\right)$ is the diameter of ablation under the action of pulse number *N*, *r*_0_ is the radius of the girdle, *P* is the average power applied to the surface of the material, and ${\;F}_{th}\left(N\right)$ is the damage threshold at the corresponding number of pulses.

For this purpose, multi-pulse laser process experiments were conducted in this paper to fit the experimental data. After the sample surface was pretreated, the average power *P* was selected as 120 mW, 200 mW, 250 mW, 350 mW and 400 mW, and the average power emitted by the laser was measured and calculated by a laser power meter to ensure that the average power emitted by the laser was consistent with it, and the surface of the samples was ablated with *N* of 10, 20, 30, 50, and 80 pulses, respectively.

After the laser treatment was completed, the two-dimensional morphology of the damage in the ablation pits was observed and the damage diameter was measured, as shown in Table 1.

**Figure 4:**
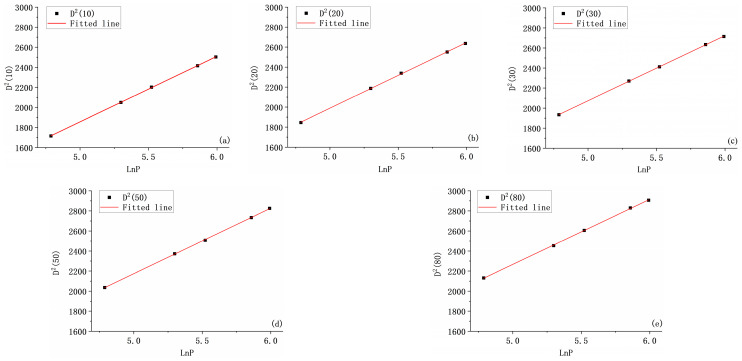
Linear fitting results of $D^{2}\left(N\right)$- lnP with pulse numbers of (**a**) 10, (**b**) 20, (**c**) 30, (**d**) 50, and (**e**) 80.

Under different average power *P* and number of pulses N, the primary equation of $D^{2}\left(N\right)$ with respect to lnP can be obtained based on the scatter ( lnP, $D^{2}\left(N\right)$), which is linearly fitted by the least squares method. As shown in Figure 4, Figure 4a–e display the fitted lines for pulse numbers *N* = 10, 20, 30, 50 and 80, respectively. The corresponding linear equations for $D^{2}\left(N\right)$ versus lnP are given by:

(4) $$\left\{\begin{array}{c} D^{2}\left(10\right)=654lnP-1416\\ D^{2}\left(20\right)=656.7lnP-1294\\ D^{2}\left(30\right)=650lnP-1176.2\\ D^{2}\left(50\right)=653.528lnP-1093.3\\ D^{2}\left(80\right)=648.73lnP-976.52 \end{array}\right.$$

Based on the fitted linear slope *k* is 2 $r_{0}^{2}$, the corresponding beam waist radius *r*_0_ can be obtained, respectively, and the average value *r*_0_ is taken to be 18 μm, with an error of less than 0.7% and minimal fluctuation, which confirms the reliability of the experimental data. When the ablation crater diameter *D*(*N*) approaches zero, the energy density at the beam center equals the damage threshold of the material, i.e., $F_{th}\left(N\right)=2P/(\pi fr_{0}^{2}).$ Therefore, by setting $D\left(N\right)=0\;$ in Equation (4), the corresponding average power *P*(*N*) at this time can be determined. Consequently, the multi-pulse damage thresholds $F_{th}\left(N\right)$ for pulse numbers *N* = 10, 20, 30, 50, 80 are calculated to be 0.17 J/cm^2^, 0.14 J/cm^2^, 0.12 J/cm^2^, 0.10 J/cm^2^, and 0.088 J/cm^2^, respectively.

Based on the relationship between $\ln\left[\begin{array}{c} NF_{th}\left(N\right) \end{array}\right]$ and lnN in Equation (2), the primary equation can be obtained by least-squares fitting according to the scatter ( lnN, $\ln\left[\begin{array}{c} NF_{th}\left(N\right) \end{array}\right]$) as:

(5) $$\ln\left[\begin{array}{c} NF_{th}\left(N\right) \end{array}\right]=0.68lnN-1.04$$

The coefficient of determination $R^{2}$ here is 0.9997, was calculated to be 0.9997, indicating an excellent fit and confirming the linear relationship between $\;\ln\left[\begin{array}{c} NF_{th}\left(N\right) \end{array}\right]$ lnN. Based on this linear regression, the accumulation coefficient *S* corresponds to the slope *k,* while the single-pulse threshold $\ln\left[\begin{array}{c} NF_{th}\left(1\right) \end{array}\right]$ is derived from the y-intercept. Consequently, the accumulation coefficient *S* was determined to be 0.68, and the single-pulse damage threshold $F_{th}\left(1\right)$ was calculated as 0.35 J/cm^2^. By substituting these values into Equation (1), the expression for the multi-pulse damage threshold of WC-6Co is established as follows:

(6) $$F_{th}\left(N\right)=0.35N^{-0.32}$$

In summary, by analyzing the cumulative effect of multi-pulse laser ablation of cemented carbide, the beam waist radius $r_{0}$ of 18 µm and the cumulative coefficient *S* = 0.68 under the experimental conditions of multi-pulse laser were obtained.

### 3.3. Thermophysical Parameters of Materials

In this study, WC-6Co cemented carbide was selected as the simulation material. Under ambient conditions, its physical properties remain relatively stable. However, high-energy pulsed laser irradiation induces drastic microscopic changes, significantly altering these thermophysical properties. Consequently, constant parameters are insufficient for solving the two-temperature model under such extreme thermal conditions. To ensure accuracy in transient thermal analysis, obtaining temperature-dependent properties is essential. Specifically, the simulation incorporates the variable heat capacity and thermal conductivity for both the electron ( $C_{e},k_{e}$) and lattice ( $C_{l},k_{l}$).

Chen et al. [23] analyzed the Boln heat transfer equation and derived a formula for the electron heat capacity expressed by Equation (7) below, which is valid for electron temperatures up to the Fermi temperature scale [24].

(7) $$C_{e}=\frac{3}{2}nk_{B}=\frac{1}{2}\pi^{2}N_{e}k_{B}\frac{T_{e}}{T_{F}}=C_{e0}T_{e}$$

Here, $N_{e}$ denotes the electron density, *k*_B_ is the Boltzmann constant, $T_{e}$ is the electron temperature, $T_{F}$ is the Fermi temperature, $C_{e0}$ is the coefficient of the electron heat capacity in J/m^3^/K^2^, and the unit of *C*_e_ is J/m^3^/K^2^. Regarding the electron density, Lin et al. [25] investigated the temperature dependence of the electron heat capacity in tungsten under strong electron–phonon non-equilibrium conditions and determined that $C_{e0}$ = 137.3 J/m^3^/K^2^. Based on this expression, the electron density of tungsten was calculated to be 2.15 × 10^29^ 1/m^3^, which is adopted in this study for the WC–6Co cemented carbide material.

The electronic thermal conductivity given by Peng et al. [9] is expressed as:

(8) $$k_{e}=k_{e0}\frac{T_{e}}{T_{l}}$$

where $k_{e0}\;$ is the electronic thermal conductivity coefficient, i.e., the thermal conductivity of the material [26], and the unit of $k_{e0}$ is W/m/K. For the thermal conductivity of WC-6Co, Wang et al. [27] found that the thermal conductivity of cemented carbide materials is affected by the WC grain size and the Co content, and the relationship between the thermal conductivity of the material and the WC grain size and the Co content is given by the equation:

(9) $$k_{e0}=154.989-87.7459*f_{Co}-65.8092*\left(\frac{1}{d}\right)$$

In this paper, the experimental sample of cemented carbide is WC-6Co material, in which the Co content is 6%, and the grain size of WC measured by the relevant equipment is 1.75 µm, and the calculation of $k_{e0}$ can be 108.6 W/m/K.

In this paper the lattice heat capacity of Cemented Carbide material is taken as the bulk heat capacity of the material, with the specific data derived from the basic study carried out by Lickschat P et al. [28]. For lattice thermal conductivity, the lattice thermal conductivity is considered to be 1% of the thermal conductivity of the bulk metal due to the fact that the mechanism of heat conduction in metals is mainly by electrons; the change in lattice thermal conductivity with a change in temperature is relatively small, and hence is taken as a constant [29].

Considering the lectroacoustic coupling coefficient $G_{el}$ of WC-6Co cemented carbide material, according to Lin et al. [25], they found that the predicted temperature dependence of the electron–phonon coupling of W can be related to the results of pump–probe photoemission measurements, thus indicating that the value of the electron–phonon coupling constant is in the range of (5–10) × 10^17^ W/m^3^/K. In this paper, we take the electroacoustic coupling coefficient $G_{el}$ as 1 × 10^18^ W/m^3^/K.

In this study, phase explosion is considered to be the main material removal mechanism [22], so the phase explosion temperature is an important parameter that determines the beginning of the material removal process. According to Guillaume B et al. [30] and Xu Y et al. [31], when the temperature reaches 0.9 times the critical temperature $T_{c}$, near the liquid, material will undergo large-scale nucleation, and when the size of the bubble nucleation reaches a certain level, the bubble continues to expand, and ultimately an explosion occurs, so the gas and the liquid material is ejected at the same time, a process known as “phase explosion”. “Peng L et al. [9] proposed a lattice deformation scheme based on energy conservation. The program will be defined as 0.9 times the material etching temperature of the critical temperature; here, it will be 0.9 ${\;T}_{c}$ defined as the phase explosion temperature $T_{exp}$, where $T_{c}$ is the critical point temperature of the material. For the material critical point temperature $T_{c}$ selection, this paper, based on Sidney Blairs et al. [32], listed the critical point temperature of the relevant metal W, and selected 20,447 K as the critical point temperature of the material studied in this paper.

The absorption of laser energy is critically dependent on the material’s optical reflectivity (*R*). For the initial surface, Lickschat et al. [28] measured the reflectivity of cemented carbide using ellipsometry under normal incidence, reporting a value of 0.46. During the laser ablation process, melt deposition and the presence of a heat-affected zone lead to the formation of a tungsten carbide (WC) layer on the surface. The presence of this WC layer directly alters the material’s absorption of laser energy, resulting in a change in reflectivity. However, measuring transient reflectivity during femtosecond ablation presents significant experimental challenges. According to Hu et al. [33], the reflectivity of WC typically ranges from 0.7 to 0.9. In this study, iterative calibration was performed within this theoretical range. By comparing the simulation results with experimental data across various laser energies, it was determined that a reflectivity of 0.88 yielded the best agreement with the experimental results. Consequently, in the multi-pulse laser model, the reflectivity was set to 0.46 for the first pulse and updated to 0.88 for all subsequent pulses.

**Figure 5:**
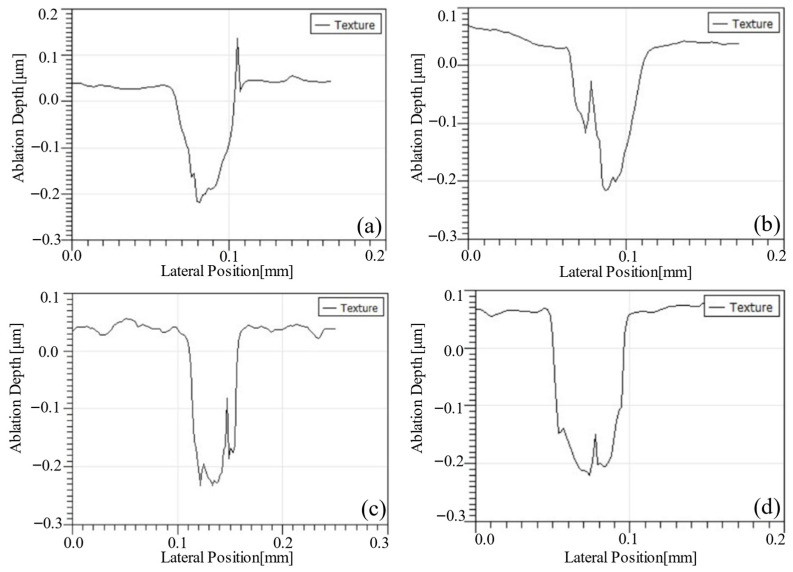
Cross-sectional contours of the crater under different average power effects. (**a**) P = 20 mW, *N* = 5; (**b**) P = 25 mW, *N* = 5; (**c**) P = 30 mW, *N* = 5; (**d**) P = 20 mW, *N* = 5.

For the absorption coefficient required in the simulation, ablation experiments were conducted with average laser powers of 20 mW, 25 mW, 35 mW, and 40 mW, using five pulses in each case. The corresponding cross-sectional profiles are shown in Figure 5a–d, which represent average powers of 20 mW, 25 mW, 35 mW, and 40 mW, respectively.

From these measurements, ablation depths of 255.3 nm, 260.1 nm, 273.4 nm, and 287.8 nm were obtained for five pulses at average powers of 20 mW, 25 mW, 35 mW, and 40 mW, respectively.

According to Urbina et al. [14], the ablation depth *d* for *N* number of pulses on the same spot is defined by Equation (10).

(10) $$d=N\cdot 1/\alpha \cdot ln\frac{F_{0}}{F_{th}(N)}$$

*α* is the absorption coefficient.

Since the ablation depth under the above pulse number and average power is known and the ablation threshold $F_{th}\left(5\right)$ under the corresponding pulse number can be obtained by combining Equation $\left(6\right)$ as 0.21 J/cm^2^, the absorption coefficient can be inversely derived for each average power, and in order to eliminate experimental errors, an average is taken. The absorption coefficient under the experimental conditions of the multi-pulse laser is obtained to be 6.12 × 10^5^ cm^−1^.

The thermophysical parameters for the numerical calculation of the two-temperature model based on WC-6Co material using COMSOL Multiphysics 5.6 software are shown in Table 2.

### 3.4. Two-Temperature Model

Femtosecond laser acting on cemented carbide can heat it up rapidly, but its internal free electron heat capacity is much smaller than that of the lattice, so the temperature of the electron and the lattice is not consistent at the early stage of irradiation, which puts it in a non-thermal equilibrium state during the irradiation process. At present, many results have been achieved in the study of ultrafast laser–matter interaction based on the two-temperature equation, and the nonlinear equation of the two-step heat conduction model with two temperatures is as follows [9,34]:

(11) $$C_{e}\frac{\partial T_{e}}{\partial t}=\frac{\partial }{\partial r}\left(k_{e}\frac{\partial T_{e}}{\partial r}\right)-G_{el}\left(T_{e}-T_{l}\right)+S\left(r,t\right)$$

(12) $$C_{l}\frac{\partial T_{l}}{\partial t}=\frac{\partial }{\partial r}\left(k_{l}\frac{\partial T_{l}}{\partial r}\right)+G_{el}\left(T_{e}-T_{l}\right)$$

where $S\left(r,t\right)$ denotes the heat source term, and $T_{e}$ and $T_{l}$ are the electron and lattice temperatures (K), respectively.

### 3.5. Boundary Condition Setting

In the simulation study of this paper, the boundary conditions are set as follows:

To simulate the common indoor temperature, the initial values of the temperature of the material and the environment are set to 293.15 K, i.e., 20 °C.

Therefore, to simplify the model, energy diffusion and refraction are neglected, and the total energy within the ablation crater is approximated as the superposition of the single-pulse energy and the cumulative energy (i.e., the product of the single-pulse energy and the number of pulses). Accordingly, the laser heat source at a depth *y* after the *N*th pulse can be expressed as [17]:

(13) $$S\left(r,t\right)=\left(1R\right)\alpha \frac{2F_{0}}{\tau }\left[{\mathrm{Gaussian}\;}_{\mathrm{space}}\left(r\right)\right]\left[{\mathrm{Gaussian}\;}_{\mathrm{time}}\left(t\right)\right]\left[L\left(x,y\right)\right]N^{1-S}$$

where *R* denotes the reflectivity of the laser on the material surface, *F*_0_ denotes the laser energy density (J/cm^2^), *τ* is the pulse width (fs), *α* is the absorption coefficient, and *N* is the number of laser pulses.

${Gaussian}_{space}\left(r\right)$ and ${Gaussian}_{time}\left(t\right)$ denote the Gaussian distribution that the laser energy obeys in the space and time domains, respectively, and are given by Equations (14) and (15):

(14) $${Gaussian}_{space}\left(r\right)=\exp\left(-2\frac{r^{2}}{r_{0}^{2}}\right)$$

(15) $${Gaussian}_{time}\left(t\right)=\sqrt{\frac{4ln2}{\pi }}\exp\left(-4\ln2\left(\frac{{\left(t-2\tau \right)}^{2}}{\tau^{2}}\right)\right)$$

where *r* is the distance from the beam center, i.e., $r=\sqrt{{\left(x-x_{0}\right)}^{2}+{\left(y-y_{0}\right)}^{2}}$, and *r*_0_ is the beam waist spot radius.

The term $L\left(x,y\right)$ in Equation (13) describes the exponential attenuation of laser energy along the depth direction, governed by the standard Beer–Lambert law ( $L=exp\left(-\alpha d\right)$), where α represents the material’s absorption coefficient and d is the laser propagation distance within the material. In this model, the laser ablation profile is approximated as a parabolic shape. Consequently, the instantaneous surface profile is described by the function $y_{surf}(x)=ax^{2}+bx+c$. The coefficients a, b, and c are the coefficients of the fitted relational equation for the ablated crater surface after the last laser pulse acts on the material surface. Therefore, for any point $\left(x,y\right)\;$ inside the material, the effective propagation distance (depth) d is defined as the vertical distance from the current surface to that point, given by $d=y_{surf}(x)-y=\left(ax^{2}+bx+c\right)-y$. Substituting this geometric relationship into the Beer–Lambert law yields Equation (16):

(16) $$L\left(x,y\right)=exp\left(-\alpha \left(\left(ax^{2}+bx+c\right)-y\right)\right)$$

It should be noted that, when establishing a complex coupling model for femtosecond laser ablation of gear tooth surfaces to address the influence of variable defocusing effects on morphology and ablation dimensions, Ming et al. [18] considered the impact of crater depth on the laser focusing radius. Their theoretical calculations revealed that in precision machining, the nanoscale material removal has a negligible effect on the focusing radius—for instance, the radius only changes by 0.07 µm over a propagation distance of 20 µm. Consequently, to improve simulation efficiency, the influence of propagation distance on the spot radius was neglected.

In order to realize the continuous simulation of the simulation model for multiple pulses, this paper utilizes the *floor* function and the *if* function to transform the laser heat source under multiple pulses to realize the automatic updating of the laser heat source under different numbers of pulses, and this paper describes the change in the laser heat source based on Equation (13) by taking three consecutive pulses as an example, as shown in Equations (17) and (18) below.

(17) $$L\left(x,y\right)=if\left(\begin{array}{c} floor\left(\frac{t}{T_{t}}\right)==2,{L\left(x,y\right)}_{3},\\ if\left(\begin{array}{c} floor\left(\frac{t}{T_{t}}\right)==1,{L\left(x,y\right)}_{2},\\ if\left(floor\left(\frac{t}{T_{t}}\right)==0,{L\left(x,y\right)}_{1},1\right) \end{array}\right) \end{array}\right)$$

(18) $$N=floor\left(\frac{t}{T_{t}}\right)+1$$

Here, $T_{t}$ denotes the pulse period, and the floor function represents the mathematical floor operation, which rounds a value down to the nearest integer toward negative infinity. ${L\left(x,y\right)}_{3}$ and ${L\left(x,y\right)}_{2}$ correspond to the fitted crater profiles resulting from the second and first laser pulses, respectively, with energy attenuation starting from these surfaces, as defined in Equation (16). ${L\left(x,y\right)}_{1}$ represents the attenuation term for the first pulse, with the attenuation beginning at the top surface of the model. Based on this definition of the laser heat source, when the first pulse begins, $floor\left(\frac{t}{T_{t}}\right)$=0, $L\left(x,y\right)$ = ${\;L\left(x,y\right)}_{1}$, and N=1, indicating that the laser acts on the top surface of the material; when the second pulse begins, $floor\left(\frac{t}{T_{t}}\right)$ = 1, $L\left(x,y\right)$ = ${L\left(x,y\right)}_{2}$, and N=2, meaning the laser interacts with the surface of the crater generated by the first pulse; and when the third pulse begins, $floor\left(\frac{t}{T_{t}}\right)$ = 2, $L\left(x,y\right)$ = ${L\left(x,y\right)}_{3}$, and N=3, meaning the laser acts on the surface of the crater formed by the second pulse. In this way, a continuous simulation of the multi-pulse laser ablation process on the material surface is realized.

When a material is irradiated by a laser, the absorbed heat is consumed mainly by thermal convection, so thermal radiation and thermal convection with air are ignored. Thermal convection occurs when part of the material reaches the phase explosion point. The convective heat flux can be expressed as:

(19) $$q_{1}=h\times \left(T-T_{l}\right)$$

(20) $$h=rm1(\left(T_{l}-T_{exp}\right)\left[1/K\right])[W/m^{2}/K]$$

Here, ${\;T}_{exp}$ denotes the phase explosion temperature of the cemented carbide. The term h represents an effective heat transfer coefficient modeled as a step function, utilizing the built-in COMSOL ramp function ( rm1). To ensure the timely simulation of material removal, the slope of this ramp function was set to a sufficiently high value of 1 × 10^8^. The ablation heat flux $q_{1}$ accounts for the energy loss due to ablation. Specifically, when the lattice temperature $T_{l}$ exceeds the critical threshold $T_{exp}\;$ (typically defined as 0.9 × $T_{c}$), material removal is initiated.

(1) For the top surface, the boundary evolves dynamically due to the material ablation process. As the material is removed, the thermal energy contained within the ablated volume is carried away, acting as the primary heat dissipation mechanism. Due to the extremely short laser–material interaction time, heat losses via convection and radiation are negligible compared to the vaporization energy. Therefore, no additional thermal boundary conditions (e.g., convective or radiative heat flux) were imposed on the top surface.
(2) Adiabatic (zero-flux) boundary conditions are applied to the lateral edges and the bottom of the geometric model, assuming no heat dissipation through these boundaries.

**Figure 6:**
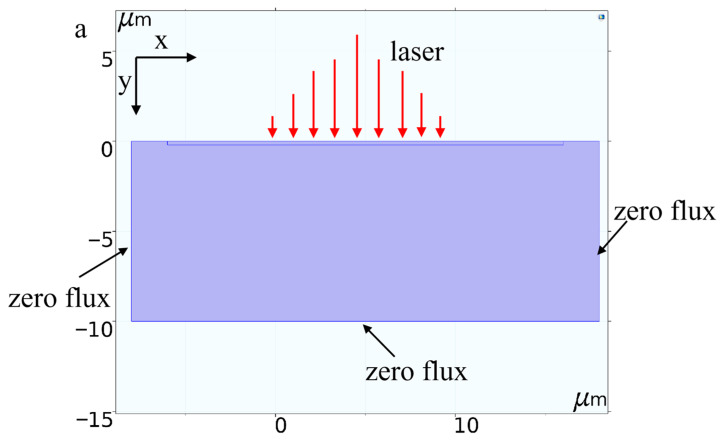
2D Model Boundary Condition Setting.

The above boundary conditions are shown in Figure 6. The simulation assumes a vertically incident laser beam centered at (*x* = 5 µm, *y* = 0 µm) acting on the cemented carbide specimen.

### 3.6. Material Removal Modeling

Based on the above discussion, the solid heat transfer module (ht) in the simulation software can be utilized to obtain the electron–lattice temperature variation law evolving with time; however, deformation geometry interface (dg) addition is required if the dynamic material removal process is to be implemented in the simulation model. In order to move the surface boundary under specific conditions, a velocity term has to be applied at the same time. The speed of the mesh deformation is determined by the removal theory at the time the laser acts on the material.

According to Nedialkov et al. [35], phase explosion is the primary mechanism for material removal under ultrashort pulsed laser irradiation. To simulate this, the Deformed Geometry (dg) interface was employed to model transient material removal via mesh deformation. When the lattice temperature reaches the phase explosion threshold (0.9 × *T_c_*), ablation-induced energy loss initially causes the lattice temperature to decrease. However, due to the continuous energy transfer from the electron–lattice interaction, the temperature is dynamically maintained near the phase explosion threshold, eventually dropping to room temperature. Based on the surface energy balance, the normal deformation velocity $\nu_{deform}$ of the lattice is shown in Equation (21) [9]:

(21) $$\nu_{deform}=\frac{q_{1}}{\rho \cdot H_{\nu }}$$

where $H_{\nu }\;$ is the latent heat of evaporation. The effect of this velocity enables the characterization of the deepening process of the crater during laser removal of the material, taking into account the energy dissipation due to the ablative removal of the material, so that the temperature field distribution and the ablation morphology of the sample surface under the action of the femtosecond laser can be obtained in real time.

### 3.7. Model Simulation Results

**Figure 7:**
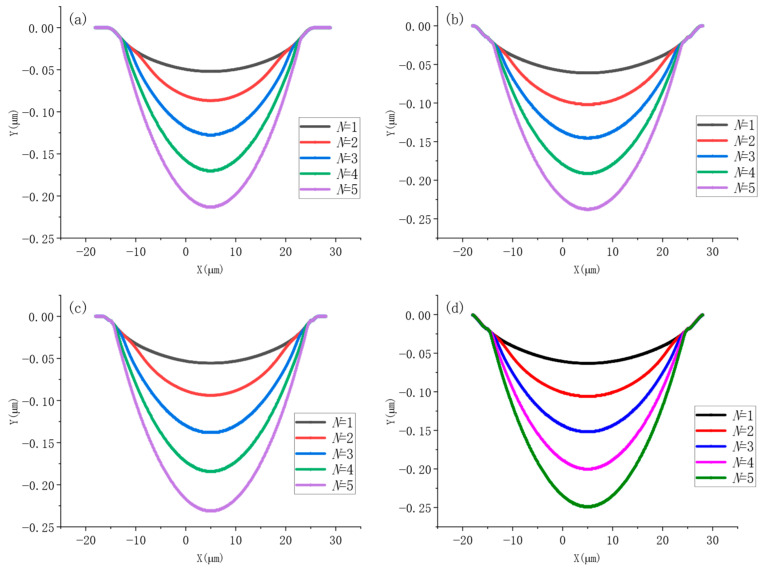
Variation in ablation pits with increasing number of pulses for different average power effects. (**a**) 20 mW; (**b**) 25 mW; (**c**) 35 mW; (**d**) 40 mW.

In the simulation model, a pulse duration of $T_{p}$ = 400 fs, a focused spot radius of $r_{0}$ = 18 µm, and a repetition rate of f=1 kHz were used. Four different average laser powers of *P* = 20 mW, 25 mW, 35 mW and 40 mW were chosen to ablate the carbide material for five pulses, and the morphology of the crater after the action of each pulse is shown in Figure 7.

**Figure 8:**
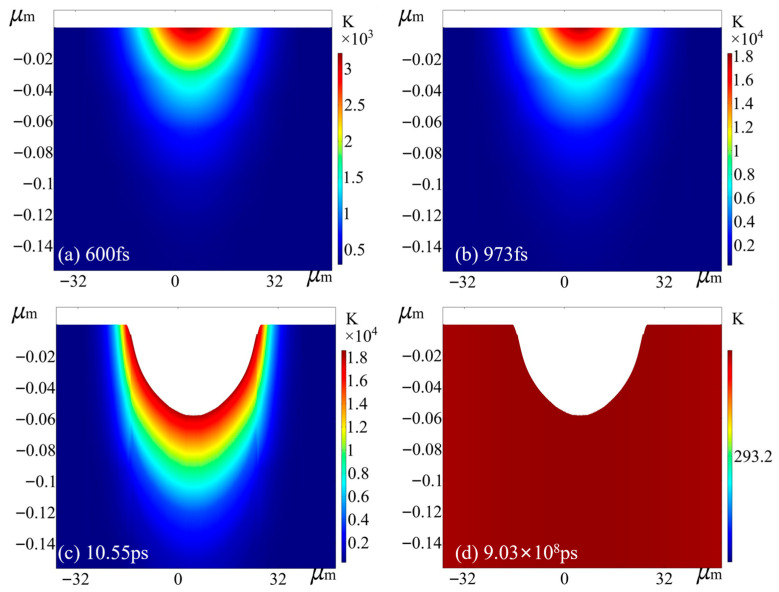
Temperature field distribution at different moments during the first pulse of laser action with P = 25 mW.

Although the thermal evolution was analyzed for all experimental power settings, the case of P = 25 mW is selected here for detailed illustration. This specific power level exhibits a deviation between simulation and experiment that lies between the minimum and maximum extremes observed, serving as a balanced and representative example to demonstrate the general ablation mechanism. Taking the multi-pulse femtosecond laser irradiation at an average power of P = 25 mW and a fluence of F_0_ = 4.91 J/cm^2^ as a representative example, the simulation results of the ablation process are illustrated. As shown in Figure 8a, at t = 600 fs, the lattice temperature reaches approximately 3212 K. Since this value is significantly lower than the phase explosion threshold of 18,402 K, no ablation marks appear on the surface. As shown in Figure 8b, at t = 973 fs, the lattice temperature rises to approximately 18,402 K due to strong electron–phonon coupling. At this point, geometric deformation occurs in the model, marking the onset of the material removal phase. Subsequently, as shown in Figure 8c, at t = 10.55 ps, a deeper and wider crater is formed. At this stage, the lattice temperature drops below the phase explosion threshold, leading to the cessation of material ablation. Figure 8d illustrates the thermal recovery of the material prior to the arrival of the subsequent pulse. Considering a laser repetition rate of 1 kHz (corresponding to a pulse interval of 1 × 10^9^ ps), the electron and lattice temperatures decrease synchronously and eventually cool to 293.41 K. Since this value is nearly identical to the ambient temperature of 293.15 K, it indicates that inter-pulse heat accumulation is effectively avoided. Following single-pulse irradiation, a crater with a depth of 55.6 nm and a diameter of 42.8 μm is formed. In this study, the crater diameter for multi-pulse ablation is quantitatively characterized at a reference depth of 0.1 nm [9].

When the simulation enters the second pulse cycle, the laser heat source automatically updates according to Equations (17) and (18) to reflect the cumulative energy distribution within the crater formed by the second pulse, with the energy decaying downward from the surface created by the first pulse ablation. Under laser irradiation, the lattice temperature at the center of the laser spot on the sample rises to approximately 3010 K at 600 fs, without inducing ablation. The temperature increase is not proportional to the higher laser energy density, primarily because the WC layer formed after the first pulse increases the surface reflectivity, resulting in reduced energy absorption compared to the first pulse. At around 1130 fs, the lattice temperature further rises to approximately 18,402 K, initiating material removal, which then continues at a relatively stable temperature. Compared to the first pulse, it takes longer for the second pulse to reach the phase explosion temperature. As time progresses, the lattice temperature drops below the phase explosion threshold at approximately 10.55 ps, marking the end of the ablation. Prior to the arrival of the next pulse, both lattice and electron temperatures gradually decrease to 293.53 K, closely approaching room temperature. After two laser pulses, the geometric model exhibits a crater with a depth of 93.8 nm and a diameter of 42.8 µm.

## 4. Simulation Results Analysis and Discussion

### 4.1. Experimental Validation of the Simulation Model

**Figure 9:**
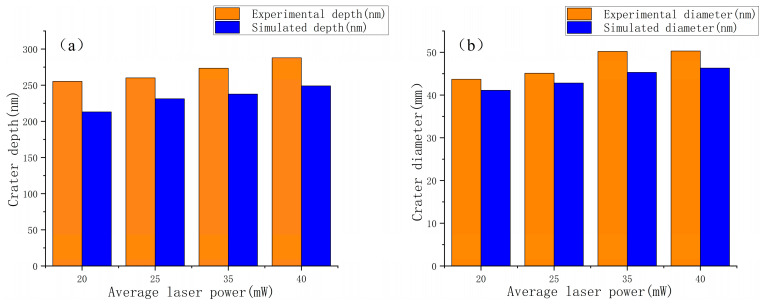
Comparison of experimental and simulated crater results at different average powers under multi-pulse laser ablation. (**a**) Crater depth; (**b**) Crater diameter.

Based on the experimental results obtained from experimental ablation with average powers of 20 mW, 25 mW, 35 mW and 40 mW, respectively, and the number of pulses of 5, a comparison of experimental and simulation results was carried out, as shown in Figure 9, which shows that the experiments and simulations are very close to each other in terms of the diameter and depth, with an average error of 7.2% and 13.6%, respectively, which verifies the accuracy of the multi-pulse laser simulation model.

**Figure 10:**
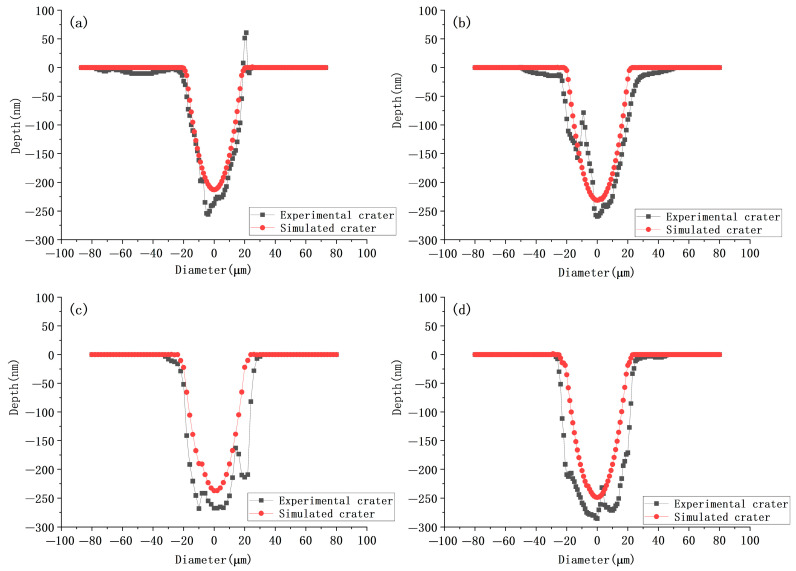
Comparison of experimental and simulated morphology under different average power effects. (**a**) 20 mW; (**b**) 25 mW; (**c**) 35 mW; (**d**) 40 mW.

In terms of morphology, a quantitative comparison between the simulated and experimental profiles is presented in Figure 10. As shown in Figure 10a,b, the simulated morphology at lower average powers (20 mW and 25 mW) exhibits excellent agreement with the experimental data, with the cross-sectional profiles overlapping significantly. However, for the higher average powers of 35 mW and 40 mW (Figure 10c,d), a noticeable deviation in ablation depth is observed, where the experimental depth exceeds the simulated depth by approximately 10–15%. Despite this vertical discrepancy, the geometric characteristics of the crater walls remain highly consistent. To quantify this, the wall inclination angles were estimated based on the steepest gradient of the profiles. For the 35 mW case (Figure 10c), the calculated physical inclination angle is approximately 1.45° for the simulation and 1.50° for the experiment, resulting in a negligible deviation of only 0.05°. Similarly, for the 40 mW case (Figure 10d), although the depth deviation increases, the wall profiles maintain good parallelism. The estimated inclination angle is 1.48° for the simulation and 1.56° for the experiment, corresponding to a deviation of approximately 0.08°. This detailed analysis indicates that across all power settings, the maximum deviation in wall inclination remains below 0.1°. It confirms that while the model may slightly underestimate the peak ablation depth at higher powers due to complex nonlinear absorption effects, it accurately predicts the lateral expansion and the taper of the ablation crater with high geometric fidelity. It is evident that both the experimental wall inclination angle and crater depth are slightly larger than the simulation results; however, the overall values remain very close. The observed deviations may be attributed to the limitations of the thermodynamic parameters used in the COMSOL model. Additionally, factors such as temperature and surface roughness also significantly influence the absorptivity of the metal [36]. The reflectivity R = 0.46 for the first pulse and R = 0.88 for the subsequent pulses selected in the simulation model of this paper may be due to the fact that the material’s absorption of the laser energy is too small, resulting in a small depth of ablation; in addition, the influence of hydrodynamics was not taken into account in the simulation, which may also be the reason for the existence of the error [37]. In this paper, the material removal is realized by using the deformation geometry module, which is to deform the mesh instead of removing the material, instead of letting the material really disappear, which may also lead to a large deviation from the experimental data.

For the studied average power range, the bottom of the experimental ablation crater generally has irregular undulating morphology, which leads to its deviation from the simulated morphology and the overall trend, which may be due to the significant difference in the thermophysical properties of tungsten carbide and cobalt composing the WC-Co. The Co bonding phase will preferentially melt or evaporate under the laser action due to its lower melting point, which results in the loss of the support of WC particles to be dislodged or redistributed, the formation of the uneven bottom of the crater, and the insufficient flow of melt. Uneven crater bottom, as well as the melt flow, is not sufficient; after the laser action, the molten Co and WC flow is driven by surface tension, but the rapid cooling of the femtosecond laser may cause the melt to solidify without sufficient spreading, forming an uneven topography. The subsequent distribution of the laser ablates the material on the uneven topography, and the final irregular undulating topography is formed under the cumulative effect. For the P = 20 mW laser, the bumps on the edge of the experimental pits may be due to the extremely high temperature generated at the moment of laser ablation, which melts or even vaporizes the material, and the molten material splashes outward under the action of the high-pressure plasma shockwave, where part of the molten material is not completely ejected away from the processed area but splashes to the edge of the pits and solidifies rapidly to form the bumps.

### 4.2. Changing Law of Electron–Lattice Temperature in Multi-Pulse Ablation Process

**Figure 11:**
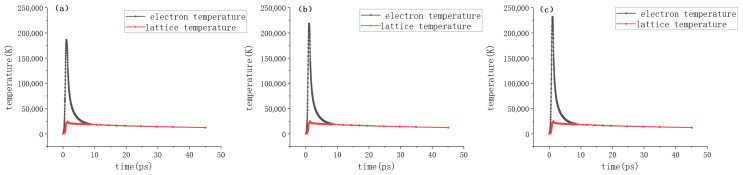
Temporal evolution of electron and lattice temperatures during the first pulse at different average powers. (**a**) P = 25 mW; (**b**) P = 35 mW; (**c**) P = 40 mW.

In this simulation, the temporal evolution of electron and lattice temperatures during multi-pulse laser ablation of WC-6Co was investigated. Figure 11a–c illustrate the temporal evolution of electron and lattice temperatures during the first pulse at average powers of 25 mW, 35 mW, and 40 mW, respectively. During the first pulse, the temperature rises sharply due to the intense energy deposition. Specifically, at average powers of 25 mW, 35 mW, and 40 mW, the peak electron temperatures reach 1.86 × 10^5^ K, 2.19 × 10^5^ K, 2.32 × 10^5^ K, respectively. The lattice temperatures also exhibit a corresponding increase with power; at t = 600 fs, the lattice temperatures are 3212 K, 3812 K, and 4078 K for the three power levels. This comparison in Figure 11 clearly demonstrates that higher laser power leads to a more significant initial temperature rise in both subsystems.

**Figure 12:**
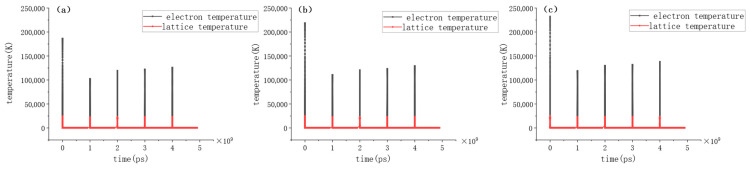
Temporal evolution of electron and lattice temperatures under five consecutive pulses at different average powers. (**a**) P = 25 mW; (**b**) P = 35 mW; (**c**) P = 40 mW.

Figure 12a–c display the temporal evolution of electron and lattice temperatures under the action of five consecutive pulses at average powers of 25 mW, 35 mW, and 40 mW, respectively. When extending the observation to multiple pulses, a distinct evolutionary trend emerges. Taking the electron temperature as an example, the peak value drops significantly in the second pulse (e.g., decreasing to 1.02 × 10^5^ K, 1.10 × 10^5^ K, and 1.19 ×10^5^ K for the respective powers). This decline is attributed to the increased surface reflectivity caused by the formation of a WC layer after the first ablation. However, from the third pulse onwards, the peak temperature gradually recovers and increases. By the fourth pulse, the peak electron temperatures rise to 1.22 × 10^5^ K, 1.23 × 10^5^ K, and 1.32 × 10^5^ K, respectively. Similarly, the lattice temperature at t = 600 fs drops in the second pulse (to 3010 K, 3730 K, and 4068 K) but subsequently rises, reaching 4639 K, 6067 K, and 6725 K by the fourth pulse. This increasing trend after the initial drop, observed in Figure 12, aligns with the heat transfer model proposing energy accumulation effects by Cai et al. [15].

Furthermore, Figure 12 also reveals the thermal state between pulses. Before the arrival of the next pulse (given the 1 kHz repetition rate), both electron and lattice temperatures have fully relaxed to thermal equilibrium, cooling to approximately 293.4 K. This value is nearly identical to the initial ambient temperature of 293.15 K, indicating that there is negligible inter-pulse heat accumulation in the bulk material.

### 4.3. Analysis of Multi-Pulse Ablation Results

**Figure 13:**
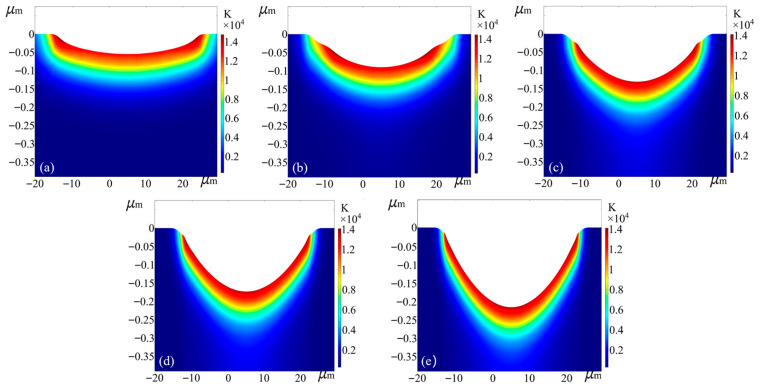
Two-dimensional morphology of the crater at P = 20 mW with different number of pulses. (**a**) *N* = 1; (**b**) *N* = 2; (**c**) *N* = 3; (**d**) *N* = 4; (**e**) *N* = 5.

In this section, the crater morphology at 40 ps for each pulse is analyzed under a laser average power of P = 20 mW. As shown in Figure 13a, after the first pulse, a crater is formed on the material surface with a depth of 52.1 nm and a diameter of 41.1 µm. As the number of pulses increases, illustrated in Figure 13b through Figure 13d, the crater depth continues to increase significantly, while the ablation diameter remains constant. Ultimately, after the fifth pulse action, as depicted in Figure 13e, the crater depth reaches 213 nm, with the ablation diameter maintaining at 41.1 µm.

**Figure 14:**
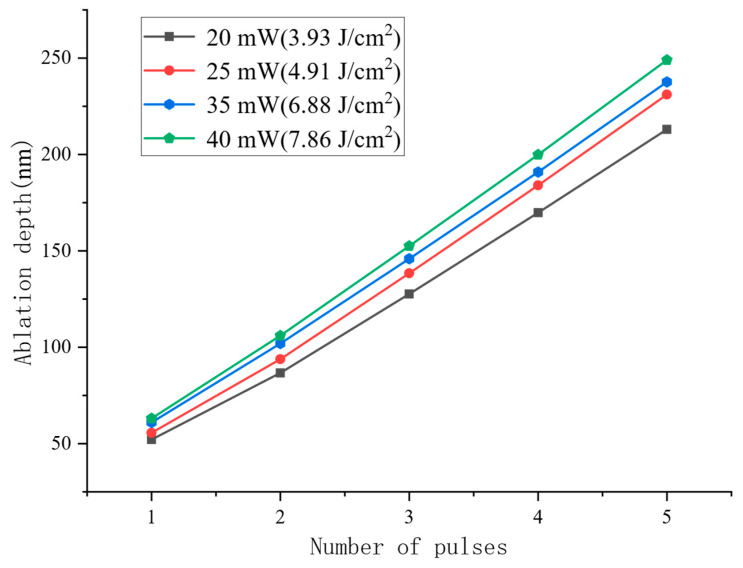
Relationship between multi-pulse ablation depth and pulse number at different average powers.

To clearly illustrate the influence of laser parameters on ablation depth, a comprehensive comparative analysis of the numerical simulation data was conducted, as shown in Figure 14. The simulation results indicate that under an average power of 20 mW, five laser pulses increased the crater depth from an initial 52.1 nm to 213 nm. When the average power was increased to 25 mW, the crater depth grew from 55.6 nm to 231.4 nm under the same number of pulses. Further increasing the power to 35 mW resulted in a depth increase from 61 nm to 237.6 nm, while at the highest average power studied, 40 mW, the five-pulse sequence extended the crater depth from 63.1 nm to 249 nm. A quantitative analysis revealed a strong positive correlation between the average laser power and the resulting ablation depth. Furthermore, in the 1–5 pulse regime, the per-pulse material removal exhibited an increasing trend, primarily due to the cumulative effect that lowers the ablation threshold with successive pulses.

## 5. Conclusions

In this study, a multi-pulse femtosecond laser ablation model for cemented carbide was developed based on the two-temperature framework, incorporating cumulative effects and an improved energy attenuation description. The main conclusions are as follows:

(1) To investigate the ablation effect of multi-pulse femtosecond lasers on cemented carbide materials, a simulation model was developed based on the two-temperature equation, incorporating the cumulative effect and an improved energy attenuation term in the laser heat source. The simulation results were validated through corresponding multi-pulse laser ablation experiments, showing good agreement with the experimental data. The average errors in crater diameter and depth between simulation and experiment were 7.2% and 13.6%, respectively, and the overall morphologies were also well matched, confirming the accuracy of the simulation model.
(2) The effects of average laser power and pulse number on the ablation process were thoroughly investigated. The second pulse exhibited a reduced peak electron temperature due to the presence of the WC layer, while subsequent pulses showed increasing electron temperatures with higher pulse counts. Additionally, under a fixed pulse number, higher average power led to higher peak electron temperatures.
(3) Temperature field analysis demonstrated that, at a repetition rate of 1 kHz, thermal accumulation between successive pulses was negligible. As the number of pulses increased, both ablation depth and diameter increased, though the diameter grew at a slower rate. Moreover, the ablation depth per pulse increased with pulse number due to the cumulative effect, indicating enhanced material removal efficiency under multi-pulse conditions.

## Figures and Tables

**Table 1:** Crater diameters for different average powers and pulse numbers.

Average Input Power	Pulse Energy	Diameters (*N* = 10)	Diameters (*N* = 20)	Diameters (*N* = 30)	Diameters (*N* = 40)	Diameters (*N* = 50)
120 mW	120 μJ	41.417 µm	42.960 µm	43.977 µm	45.125 µm	46.171 µm
200 mW	200 μJ	45.270 µm	46.773 µm	47.648 µm	48.710 µm	49.536 µm
250 mW	250 μJ	46.926 µm	48.364 µm	49.113 µm	50.068 µm	51.048 µm
350 mW	350 μJ	49.148 µm	50.504 µm	51.325 µm	52.288 µm	53.194 µm
400 mW	400 μJ	50.027 µm	51.345 µm	52.103 µm	53.158 µm	53.913 µm

**Table 2:** Thermophysical parameters of WC-6Co under single-pulse laser action.

Thermophysical Parameter	Notation	Unit	Value	Bibliography
Electron density (pure tungsten)	*N*e	m^−3^	2.15 (10^29^)	[25]
Fermi temperature (pure tungsten)	*T*_F_	K	1.066 (10)	[9]
Specific heat coefficient of electrons	*C*_e0_	J/m^3^/K^2^	137.3	[25]
Electronic thermal conductivity coefficient	*k*_e0_	W/m/K	108.6	[27]
lattice heat capacity	*C*_l_	J/kg/K	200	[28]
lattice thermal conductivity	*k*_l_	W/m/K	1.086	[29]
Critical temperature (pure tungsten)	*T*_c_	K	20447	[32]
Electron–lattice coupling coefficient	*G*_el_	W/cm^3^/K	1 (10^12^)	[25]
reflectivity	*R*	1	0.46–0.88	[28,33]
enthalpy of evaporation	*H*_v_	J/kg	3.69 (10^6^)	[28]

## Data Availability

The original contributions presented in the study are included in the article, further inquiries can be directed to the corresponding author.
